# No Metagenomic Evidence of Causative Viral Pathogens in Postencephalitic Parkinsonism Following Encephalitis Lethargica

**DOI:** 10.3390/microorganisms9081716

**Published:** 2021-08-12

**Authors:** Dániel Cadar, Kurt A. Jellinger, Peter Riederer, Sabrina Strobel, Camelia-Maria Monoranu, Dennis Tappe

**Affiliations:** 1 Bernhard Nocht Institute for Tropical Medicine, 20359 Hamburg, Germany; danielcadar@gmail.com; 2 Institute of Clinical Neurobiology, 1090 Vienna, Austria; kurt.jellinger@univie.ac.at; 3 Center of Mental Health, Department of Psychiatry, Psychosomatics and Psychotherapy, University Hospital of Würzburg, 97080 Würzburg, Germany; peter.riederer@mail.uni-wuerzburg.de; 4 Department of Neuropathology, Institute for Pathology, University of Würzburg, 97080 Würzburg, Germany; sabrina.strobel@uni-wuerzburg.de (S.S.); camelia-maria.monoranu@uni-wuerzburg.de (C.-M.M.)

**Keywords:** postencephalitic parkinsonism, encephalitis lethargica, von Economo, metagenomics, neuropathology, tauopathy

## Abstract

Postencephalitic parkinsonism (PEP) is a disease of unknown etiology and pathophysiology following encephalitis lethargica (EL), an acute-onset polioencephalitis of cryptic cause in the 1920s. PEP is a tauopathy with multisystem neuronal loss and gliosis, clinically characterized by bradykinesia, rigidity, rest tremor, and oculogyric crises. Though a viral cause of EL is likely, past polymerase chain reaction-based investigations in the etiology of both PEP and EL were negative. PEP might be caused directly by an unknown viral pathogen or the consequence of a post-infectious immunopathology. The development of metagenomic next-generation sequencing in conjunction with bioinformatic techniques has generated a broad-range tool for the detection of unknown pathogens in the recent past. Retrospective identification and characterization of pathogens responsible for past infectious diseases can be successfully performed with formalin-fixed paraffin-embedded (FFPE) tissue samples. In this study, we analyzed 24 FFPE brain samples from six patients with PEP by unbiased metagenomic next-generation sequencing. Our results show that no evidence for the presence of a specific or putative (novel) viral pathogen was found, suggesting a likely post-infectious immune-mediated etiology of PEP.

## 1. Introduction

Encephalitis lethargica (EL, von Economo disease) was an epidemic polioencephalitis of unknown etiology with an acute-onset. The disease was responsible for more than 1 million human cases and half a million deaths during 1917–1925. To a lesser extent, the disease was reported until 1940 [1]. Approximately one-third of EL patients developed postencephalitic parkinsonism (PEP), clinically characterized by bradykinesia, rigidity, rest tremor, and—almost pathognomonic—oculogyric crises. PEP ensued directly after EL (or weeks to many years later) [1], sometimes in a complex relationship [2,3]. PEP is a tauopathy with multisystem neuronal loss and gliosis accompanied by widespread neurofibrillary lesions, consisting of 3- and 4-repeat (3R and 4R) hyperphosphorylated tau isoforms [4]. PEP does not show alpha-synuclein pathology and, thus, differs from idiopathic Parkinson’s disease [5,6]. In the 1920s–1930s, PEP was the most frequent form of parkinsonism. Despite the frequency of EL and PEP, their etiology and pathogenesis are unknown. A viral pathogen or a post-infectious immune-mediated mechanism is probably the cause of PEP, whereas EL is likely of direct viral etiology [1]. As a wide range of viruses is capable of causing acute or delayed neuropathology in humans [7], including, as recently shown, the bornaviruses [8,9], the list of candidate pathogens for PEP and EL is extensive [10,11].

Often, the retrospective identification of pathogens from past outbreaks is limited by the absence of stored serum or cerebrospinal fluid samples [12]. However, formalin-fixed paraffin-embedded (FFPE) samples sometimes remain in pathology archives from such epidemics, outbreaks, case series, or single cases of unknown etiology. FFPE tissues have been used increasingly for successful retrospective pathogen discovery by molecular techniques [13,14]. However, the past application of polymerase chain reaction assays for a possible influenza etiology of EL or PEP was negative [15,16].

Here, we analyzed different brain areas from six patients with PEP after EL for a possible infectious etiology by unbiased metagenomic next-generation sequencing (NGS). 

## 2. Materials and Methods

### 2.1. Patients and Available Materials

**Figure 1:**
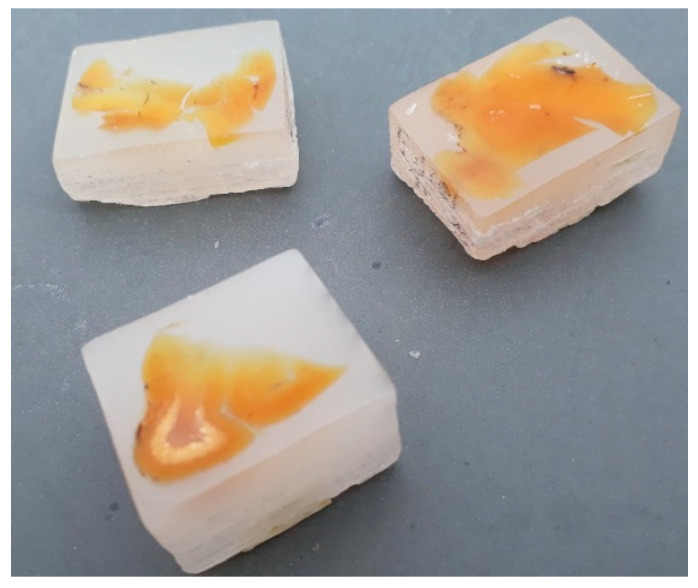
Formalin-fixed paraffin-embedded brain tissue specimens from patients with postencephalitic parkinsonism.

From autopsy cases of six Caucasian patients (two males, aged 62 and 66 years; four females, aged 51–77 years), FFPE brain tissue blocks were available from 2 to 8 different brain regions (Table 1, Figure 1) for this study. PEP had been diagnosed clinically and had followed 10–44 years (median 21 years) after EL. Tauopathy had been demonstrated in four individuals (patients 1–4) in a clinicopathological case series before [5]. Tissue blocks were stored for 38–62 years at room temperature before the current analysis. Ethical clearance was obtained from the local ethics board (Medical Board of Hamburg, no. PV5616; 15 July 2019).

### 2.2. Immunohistochemistry from Archived Brain Tissues

Immunohistochemistry for phospho-tau and alpha-synuclein was performed from all samples. For phospho-tau detection, the PHF Tau AT8 antibody (Thermo Fisher Scientific, Bremen, Germany) was used, for alpha-synuclein detection, the Syn303 antibody (BioLegend, San Diego, CA, USA). After pretreatment with citrate (pH 6) and blocking of endogenous peroxidase, the tissue sections were incubated overnight at room temperature with the respective antibody 1:100 in Antibody Diluent Solution (Zytomed Systems, Berlin, Germany).

This step was followed by incubation with the DCS-AEC 2 Component Detection Kit and 3-amino-9-ethylcarbazole substrate (DCS, Hamburg, Germany) for immunoperoxidase staining. As positive control for tau pathology detection, a case with Alzheimer’s dementia was used, and as positive control for alpha-synuclein detection a case with Parkinson’s disease.

### 2.3. Sequence-Independent Amplification and Next-Generation Sequencing of FFPE Tissue Samples

After microtome and blade decontamination using RNase and DNase away (Thermo Scientific), 10-µm-thick sections from FFPE tissue blocks were used. Following deparaffinization (Deparaffinization Solution, Qiagen, Hilden, Germany), RNA was extracted from each of the 24 blocks using the MagMAX™ FFPE DNA/RNA Ultra Kit (Applied Biosystems, Austin, TX, USA), according to the instructions of the manufacturer for RNA and separately for DNA extraction. In order to evaluate the quality and integrity of the extracted DNA and RNA, we have quantified the DNA and RNA by using Qubit™ DNA/RNA HS Assay Kit (Thermo Fisher Scientific, Austin, TX, USA) and the RNA degradation by RNA Integrity Number (RIN) using Agilent 2100 Bioanalyzer (Agilent, Santa Clara, CA, USA). A host nucleic acid depletion step with QIAseq FastSelect-rRNA HMR Kit (Qiagen, Hilden, Germany) was added. RNA extracts underwent random amplification by RT-PCR, followed by an enhancement amplification PCR step with the same random primers [17]. The obtained cDNA from RNA fraction has been mixed with the extracted DAN and subjected to library preparation using a QIAseq FX DNA Library Kit (Qiagen, Hilden, Germany). Normalized samples were pooled and sequenced on a NextSeq 2000 platform using the 200-cycle (2 × 100 bp paired-end) NextSeq2000 P2 reagent kit (Illumina, San Diego, CA, USA). All reads were checked, trimmed and filtered to remove polyclonal and low-quality reads (<50 bases) using the CLC Workbench (Qiagen, Hilden, Germany). Filtered reads were de novo assembled with Geneious v9.1.8 (Biomatters, Auckland, New Zealand) and homology search was performed using BLASTX (blast.ncbi.nlm.nih.gov; accessed on May 2021) against GenBank with an E-value cutoff of 0.001. The outputs were analyzed by the metagenomic analyzer software MEGAN (https://software-ab.informatik.uni-tuebingen.de/download/megan6/welcome.html; accessed on 31 May 2021) according to their taxonomy. For extraction and metagenomics control, a FFPE brain sample of an HIV-positive patient from 1994 and sterile water were used in parallel.

## 3. Results

### 3.1. Immunohistochemistry for Postencephalitic Parkinsonism (PEP)

**Figure 2:**
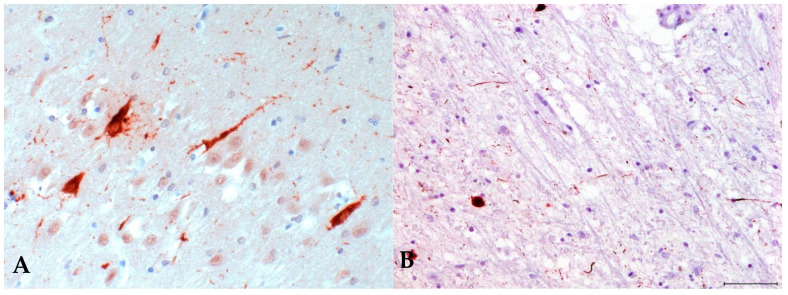
Neuropathology of postencephalitic parkinsonism. (**A**) Hyperphosphorylated tau is demonstrated as flame-shaped intraneuronal neurofibrillary tangles and also seen in the neuropil. Immunoperoxidase stain with hematoxylin counterstain. Midbrain, 200-fold magnification. (**B**) Hyperphosphorylated tau in globose neurofibrillary tangles and in neuropil threads. Immunoperoxidase stain with hematoxylin counterstain. Midbrain, 100-fold magnification.

Immunohistochemical analysis of tissue from different brain regions detected typical tau-positive lesions in all samples (Figure 2A,B) while alpha-synuclein pathology was absent (not shown); thus, confirming the clinical diagnosis of PEP. There was widespread gliosis and no inflammation. 

### 3.2. Identification of Viral Sequences in FFPE Samples Using NGS

**Figure 3:**
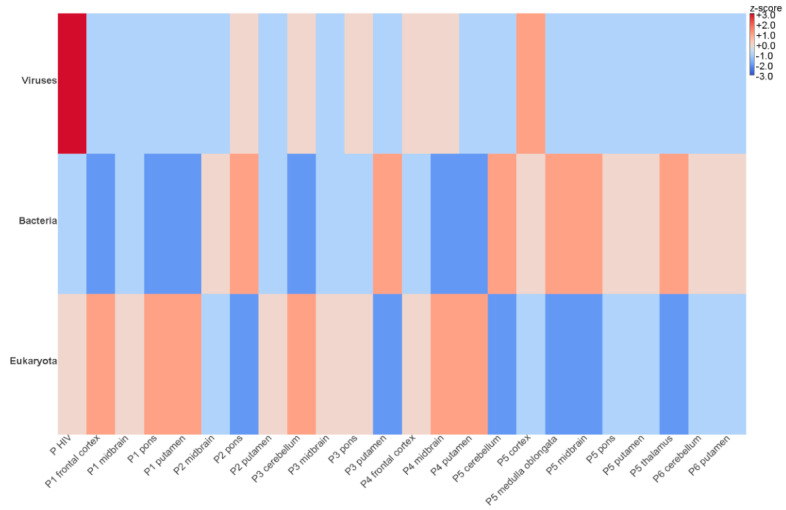
Heat map visualizing the abundances of eukaryotic, bacterial, and viral reads in each brain area from six patients with PEP. The brain regions of the PEP patients are listed in the bottom text row. The heat map is color-coded based on row z-scores; P HIV—brain from HIV infected patient used as positive control.

**Figure 4:**
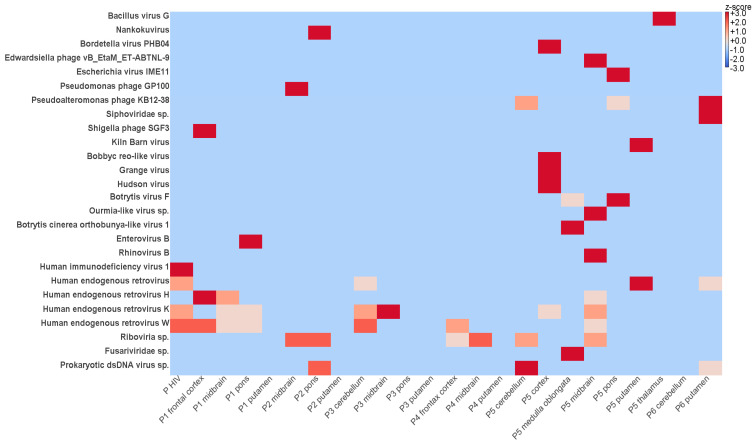
Heat map visualizing the most abundant virus species in each brain area from six patients with PEP. The brain regions are listed on the X axis. The virus species are presented on the Y axis. The map is color-coded based on row z-scores; P HIV—brain from HIV infected patient used as positive control.

By metatranscriptomic NGS, between 7,959,775 and 13,412,314 reads were obtained after trimming and quality check and further analyzed by an in-house virus discovery pipeline (Table 2). Most of the reads detected in the FFPE brain tissues were categorized as eukaryotic and bacterial sequences (Figure 3). Metatranscriptomics revealed relatively low levels of viral diversity, the most abundant viral groups detected being the *Picornaviridae, Retroviridae, Reoviridae, Fusariviridae, Botourmiaviridae* (Figure 4). Except for the human immunodeficiency virus 1 from the patient used as positive control (genomic coverage ~39%), all retrovirus reads have been categorized as endogenous retroviral sequences (Figure 4). The other viral findings including those from the *Reoviridae* have shown high similarity with viruses infecting plants and insects. Thus, the presence of these viral reads is likely due to environmental contamination.

No viruses known to be involved in encephalitis (e.g., herpesviruses, influenza viruses, enteroviruses, bornaviruses, paramyxoviruses, flaviviruses, rabies virus, human immunodeficiency virus, etc.), or highly divergent or even unknown novel viral reads in the FFPE tissue samples subjected to deep sequencing were detected using the in-house established virus discovery pipeline. Nevertheless, we were able to detect in the pons section of patient 1 a human enterovirus B75 sequences (genomic coverage ~16%) and in the midbrain section of patient 5 a rhinovirus B70 sequences (genomic coverage ~9%). However, the contigs show high similarity with recent enterovirus and rhinovirus relatives, suggesting most likely contaminations. The library obtained from the negative water control did not contain any known or novel viral reads. In contrast, HIV sequences could be retrieved from the positive control brain sample as expected (Figure 4).

## 4. Discussion

NGS, a broad-range technique for the detection and characterization of (novel) pathogens, has seen an expanding applicability including the analysis of archived FFPE tissue samples [17,18]. However, formalin fixation, which allows tissue preservation and storage at room temperature, leads to cross-linking of macromolecules including nucleic acids and complicates the extraction of intact DNA and RNA. NGS might be less sensitive than PCR from FFPE samples on some sequencing platforms. This problem might be solved by using higher sequence depths [12], but will increase the cost per sample especially in smaller studies. It was shown that specimens from a study with adenocarcinomas when stored for longer time periods had significantly lower coverage of the genetic target (6% lower per 10 years) and lower average read depth (40× lower per 10 years) [19]. However, sufficient quality and quantity of NGS data were obtained, and irrespective of storage duration, 90% of specimens provided usable NGS data [19]. 

In this study, an unbiased sequence-independent amplification in combination with NGS on FFPE tissues from patients has been applied. Despite using a high sequencing depth on 24 FFPE tissue samples from 6 patients with PEP following EL, we did not detect known or putatively novel RNA viruses with plausible encephalitic potential. Both the human rhinovirus B70 and the enterovirus B75 read in one sample each are likely contaminations as the show high sequence similarity with recent virus variants. Our virus discovery pipeline has been successfully used before with FFPE brain samples [17,20,21]. Moreover, we were recently able to recover an Ebola virus genome from a 45-year-old FFPE block using the same pipeline and also without a target enrichment approach (unpublished personal data), and the HIV sequence from a control FFPE sample stored for 27 years could also be retrieved. This does not exclude a possible failure of our method to detect viral pathogen sequences due to the advanced age and fixation of the samples, keeping in mind that possible viral pathogen nucleic acids represent only a minor fraction of recovered DNA or RNA; however, such a scenario seems rather unlikely as our pipeline was able to detect the aforementioned Ebola virus and HIV sequences. 

EL is presumably caused by a viral pathogen directly [1], and PEP might be caused by the same pathogen after EL, or, later on, by the same pathogen if it becomes persistent. However, PEP might also be the neurodegenerative result of an indirect, immune-mediated pathogen-triggered process. It was recently suggested that a viral infection of the substantia nigra predisposes for later parkinsonism [22]. PEP is associated with mortality rates of up to 40%, but residual survivors continued to live on into old age [4]. In our analysis of PEP samples many years after onset of the disease, at least no persistent pathogens were detected. This negative finding makes a hit-and-run mechanism for a pathogen causing EL followed by a post-infectious immune-mediated etiology for PEP very plausible. PEP is a multisystem tauopathy characterized by abundant neuronal loss, neurodegeneration, gliosis, and phospho-tau accumulation in neurons and glia. Phospho-tau deposits generate neurofibrillary tangles (NFT) similar to those found in Alzheimer’s dementia [4], with prominent globose NFTs (Figure 2B) in residual neurons of the substantia nigra and other brainstem nuclei [4]. NFTs are also present in the hippocampus, temporal, frontal and insular cortex in PEP [5]. The tauopathies comprise a range of neurodegenerative diseases, such as Alzheimer’s dementia, Pick’s disease, progressive supranuclear palsy, corticobasal degeneration, and others. The pathogenesis of tauopathies is unclear. Normal tau facilitates the assembly of tubulin into microtubules. Abnormally hyperphosphorylated tau induces the sequestration of normal tau in a prion-like manner [23,24]. Likely, the cytosolic soluble hyperphosphorylated form is pathogenic, and not the inert, deposited neurofibrillary phospho-tau tangle [23]. Phospho-tau accumulation in disorders considered to have another driving force than primary protein deposits are referred to as secondary tauopathies [24], which is clearly the case in PEP following EL. Tau pathology in the form of neurofibrillary tangles has also been seen after encephalitis caused by West Nile virus [25] and in subacute sclerosing panencephalitis (SSPE) due to measles virus [26]. It seems plausible that a virus-induced inflammation of the brain (as most probable etiology of EL) is a trigger that might lead to tau hyperphosphorylation in PEP, which in turn might cause neurodegeneration. An immune-triggered phospho-tau formation has recently been shown in anti-IgLON5-related tauopathy, a parasomnia with bulbar dysfunction due to an autoantibody against a neuronal cell-adhesion molecule [27]. The exact mechanism of induction of tau pathology by the antibody is so far unclear, however. Anti- IgLON5-related tauopathy is strongly associated with two specific HLA types, and different HLA types reflecting individual susceptibility or immune responses might also be responsible for the complex relationship between EL and PEP. Autoimmune phenomena of the central nervous system after viral encephalitis are further exemplified by NMDA-receptor encephalitis and other forms auf autoimmune encephalitis developing after herpes simplex virus encephalitis [28]. Unfortunately, we were not able to locate any serum or CSF samples of patients with PEP to test for autoantibodies. Recently it was shown in animal models that an activation of the NLRP3 inflammasome drives tau pathology [29]. Such a mechanism may also play a role in PEP after EL.

As a drawback of our study, besides sensitivity issues due to formalin-fixation and storage time, further limitations encompass the limited sample size and the circumstance that PEP was long-standing in several patients before death. Future investigations should thus comprise more specimens, and in earlier stages of PEP and EL.

## 5. Conclusions

Our negative unbiased sequence-independent NGS findings from multiple FFPE PEP samples support the hypothesis of a post-infectious immune-mediated etiology for PEP. However, due to the limited number of patients tested, our observations warrant further investigation. More archived patient samples, especially from early-onset PEP, but also from acute EL, are needed to shed more light on the etiology of both diseases. 

## Figures and Tables

**Table 1:** Clinical characteristics of patients with postencephalitic parkinsonism in this study.

Patient	Sex	Age at Time of Death (Year)	Onset of EL, Onset of PEP; Interval	Storage Time Until Analysis	Clinical Symptoms
1	F	66 (1979)	1939, 1951; 12 years	42 years	akinesia, rigidity, oculogyric crises, tremor, hypomimia, gait disorder
2	F	77 (1982)	1921, 1965; 44 years	39 years	akinesia, rigidity, gait disturbances
3	M	66 (1983)	1932, 1960; 28 years	38 years	akinesia, rigidity, oculogyric crises, sialorrhea, gait disorder
4	F	65 (1977)	1923, 1933; 10 years	44 years	akinesia, bradykinesia, rigidity, oculogyric crises
5	F	51 (1959)	1926, 1945; 19 years	62 years	akinesia, rigidity, oculogyric crises, hypomimia
6	M	62 (1970)	1923, 1950; 27 years	51 years	akinesia, rigidity, gait disturbances, oculogyric crises
7 *	M	unknown	N/A	27 years	N/A

* HIV control brain; EL, encephalitis lethargica; PEP, postencephalitic parkinsonism.

**Table 2:** Host and virus reads from transcriptome sequencing data of patient brain sections.

Patient	Brain Regions Available and Sequenced by NGS	Trimmed Reads	Host Reads	Viral Reads
1	frontal cortex	9,517,032	3,639,675	302
	putamen	8,920,649	3,229,635	736
	midbrain	9,718,416	3,978,842	503
	pons	8,543,882	3,252,964	725
2	putamen	9,565,917	3,170,398	831
	midbrain	8,840,396	2,086,639	1136
	pons	9,638,905	1,850,362	7613
3	cerebellum	10,901,531	4,228,111	1145
	putamen	8,132,530	2,063,999	1354
	midbrain	9,522,435	4,053,318	4406
	pons	7,959,775	3,268,825	4540
4	frontal cortex	10,557,783	4,130,625	1295
	putamen	10,221,537	4,338,290	3600
	midbrain	9,838,675	4,584,931	1256
5	cortex	8,452,734	1,802,060	3984
	thalamus	10,267,166	1,340,110	1688
	cortex	9,139,183	1,787,936	3453
	putamen	9,534,555	1,759,967	2344
	midbrain	12,018,105	2,011,537	2552
	cerebellum	11,802,862	1,490,561	2147
	pons	12,276,639	4,245,357	1198
	medulla oblongata	13,412,314	3,597,636	3849
6	putamen	9,080,742	1,510,840	1235
	cerebellum	11,276,435	1,037,770	2289
7 *	midbrain	11,345,665	1,227,560	8435

* HIV control brain
